# Controlled Human Malaria Infection of Tanzanians by Intradermal Injection of Aseptic, Purified, Cryopreserved *Plasmodium falciparum* Sporozoites

**DOI:** 10.4269/ajtmh.14-0119

**Published:** 2014-09-03

**Authors:** Seif Shekalaghe, Mastidia Rutaihwa, Peter F. Billingsley, Mwajuma Chemba, Claudia A. Daubenberger, Eric R. James, Maximillian Mpina, Omar Ali Juma, Tobias Schindler, Eric Huber, Anusha Gunasekera, Anita Manoj, Beatus Simon, Elizabeth Saverino, L. W. Preston Church, Cornelus C. Hermsen, Robert W. Sauerwein, Christopher Plowe, Meera Venkatesan, Philip Sasi, Omar Lweno, Paul Mutani, Ali Hamad, Ali Mohammed, Alwisa Urassa, Tutu Mzee, Debbie Padilla, Adam Ruben, B. Kim Lee Sim, Marcel Tanner, Salim Abdulla, Stephen L. Hoffman

**Affiliations:** Ifakara Health Institute, Bagamoyo Research and Training Centre, Bagamoyo, Tanzania; Sanaria Inc., Rockville, Maryland; Protein Potential LLC, Rockville, Maryland; Swiss Tropical and Public Health Institute, Basel, Switzerland; University of Basel, Switzerland; Radboud University Nijmegen Medical Center, Department of Medical Microbiology, Nijmegen, The Netherlands; Howard Hughes Medical Institute and Center for Vaccine Development, University of Maryland School of Medicine, Baltimore, Maryland; Muhimbili University of Health and Allied Sciences, Dar es Salaam, Tanzania

## Abstract

Controlled human malaria infection (CHMI) by mosquito bite has been used to assess anti-malaria interventions in > 1,500 volunteers since development of methods for infecting mosquitoes by feeding on *Plasmodium falciparum* (Pf) gametocyte cultures. Such CHMIs have never been used in Africa. Aseptic, purified, cryopreserved Pf sporozoites, PfSPZ Challenge, were used to infect Dutch volunteers by intradermal injection. We conducted a double-blind, placebo-controlled trial to assess safety and infectivity of PfSPZ Challenge in adult male Tanzanians. Volunteers were injected intradermally with 10,000 (*N* = 12) or 25,000 (*N* = 12) PfSPZ or normal saline (*N* = 6). PfSPZ Challenge was well tolerated and safe. Eleven of 12 and 10 of 11 subjects, who received 10,000 and 25,000 PfSPZ respectively, developed parasitemia. In 10,000 versus 25,000 PfSPZ groups geometric mean days from injection to Pf positivity by thick blood film was 15.4 versus 13.5 (*P* = 0.023). Alpha-thalassemia heterozygosity had no apparent effect on infectivity. PfSPZ Challenge was safe, well tolerated, and infectious.

## Introduction

Controlled human malaria infection (CHMI), intentional infection of subjects with malaria parasites, has been used for treating patients with syphilis^1^,^2^ and in research for nearly a century.^3^ Since the development in the 1980s of methods for infecting mosquitoes by feeding on *Plasmodium falciparum* (Pf) gametocyte cultures,^4^–^6^ CHMI has been used repeatedly and successfully in more than 1,500 volunteers in the United States and Europe.^6^–^10^ Africa suffers the most morbidity and mortality from malaria, and thus could benefit significantly by using CHMI to facilitate development of new vaccines, drugs, and diagnostics for malaria, and for understanding innate and acquired resistance to the parasites that cause malaria. However, until now such CHMIs had never been used in Africa.

There are a number of reasons why CHMI has not been established in Africa. One is that the phase 1 clinical trial facilities and teams necessary to safely and professionally carry out such trials have not been available until recently for such studies. A second reason is that from 1985 to 2010, all CHMI studies in which volunteers were infected with Pf sporozoites were conducted by exposure to the bites of Pf-infected *Anopheles* mosquitoes not native to Africa, and produced in high security facilities difficult to establish, run, and maintain in Africa. To address the first limitation, we established a phase 1 clinical trial center at the Ifakara Health Institute (IHI), Bagamoyo, Tanzania. At the same time it became possible to manufacture aseptic, purified, cryopreserved Pf sporozoites (PfSPZ) that are highly infectious, a product called PfSPZ Challenge.^11^–^13^ When young adult Dutch volunteers were injected intradermally (ID) with doses of 2,500, 10,000, or 25,000 PfSPZ (divided into two 50 μL injections), five of six volunteers developed parasitemia in all three groups, and the time from injection of PfSPZ Challenge to detection of parasites by thick blood smear was ∼13 days in all three groups. Thus, there was infection, but no dose response, presumably because increasing the dose did not increase the numbers of sporozoites that exited the skin, entered the circulation, and invaded hepatocytes.^12^

To begin the process of understanding how to use PfSPZ Challenge in Africans, we conducted a double-blind, placebo-controlled trial to assess the safety and infectivity of ID-administered PfSPZ Challenge in 30 male, highly educated, Tanzanian residents of Dar es Salaam, Tanzania, who had had minimal exposure to Pf malaria during the previous 5 years. As a bridge to the Dutch study, one group received the regimen used in one of the Dutch groups; 5,000 PfSPZ in 50 μL were injected ID into the deltoid area of both upper arms for a total of 10,000 PfSPZ.^12^ In a second group we increased the dose to 25,000 PfSPZ, and based on findings in murine model systems, which suggested ways to improve the efficiency of ID injections,^14^ we divided the total dosage into four injections of 6,250 PfSPZ, each in 10 μL.

As in Dutch^12^ and British^13^ subjects, PfSPZ Challenge was safe, well tolerated, and infectious in young adult Tanzanian males. In the bridging group the infection rate, but not the pre-patent period, was comparable to that observed for the same dose in young adult Dutch subjects. These findings provide the foundation for using CHMI with PfSPZ Challenge to assess the protective efficacy of antimalarial vaccines and drugs in Africa.

## Materials and Methods

### Study design and population.

This single center, double-blind, randomized, controlled trial was conducted in Bagamoyo, Tanzania between February and August 2012. Thirty healthy male volunteers 20 to 35 years of age were recruited from higher learning institutions in Dar es Salaam. Screening for eligibility took place at the newly established Bagamoyo Clinical Trial Unit (BCTU) of the Ifakara Health Institute (IHI). Volunteers were screened using predetermined inclusion and exclusion criteria based on clinical examinations and laboratory tests. These included medical history and physical examinations, and standard hematology, biochemistry, malaria, human immunodeficiency virus, hepatitis B and C, and sickle cell tests. In addition subjects were screened for α-thalassemia. In the initial screening α-thalassemia trait was an exclusion criterion because of a theoretical concern that these individuals might be less susceptible to Pf infection. As screening progressed it became clear that a substantial proportion of the local population was heterozygous for α-thalassemia and it would be important to include this population in the volunteer pool to understand if the heterozygous condition was more resistant to malaria infection by experimental challenge and pose an obstacle that would need to be addressed in future vaccine studies. Volunteers who indicated that they had not had an episode of documented malaria in the past 5 years were included. They also had malaria thick smears, and any subject who was positive was excluded.

#### Screening for a+ and a^0^ α-thalassemia caused by deletions.

One milliliter (1 mL) of venous blood was collected in EDTA tubes and stored at −80°C. The DNA was extracted from 100 μL of whole blood with ZR Genomic DNA-Tissue MiniPrep (ZymoResearch, Irvine, CA) according to manufacturer's recommendations. We used primers to amplify the alpha 2 globin gene as a control and the 3.7 kb, 4.2 kb, and 20.5 kb deletion junction fragments of the α-thalassemia variants that could be easily identified by size as described.^15^ The multiplex polymerase chain reaction (PCR) primers used were a 2/3.7del F, 3.7del/20.5del R, a2 R, 4.2del F, 4.2del R, and 20.5del F. The PCR reaction contained additionally 1× Q-solution 2.5 U HotStarTaq DNA polymerase in supplied reaction buffer (Qiagen, Valencia, CA) and 100 ng of genomic DNA. Reactions were carried out on a thermal cycler (Gene Amp 2700, Applied Biosystems, Foster City, CA), with an initial 15-minute denaturation at 96°C, 30 cycles of 98°C for 45 seconds, 60°C for 90 seconds, 72°C for 135 seconds, and a final extension at 72°C for 5 minutes. Following amplification, 10 μL of the product were electrophoresed through a 1.5% agarose gel with 0.6 μg/mL ethidium bromide in 1× TBE buffer first at 7 volts/cm for 1 hour followed by 3 volts/cm for an additional 2 hours. The gel was visualized on an UV transilluminator. The wild-type aa/aa loci yielded a PCR product of 1,800 base pair (bp), whereas the 3.7 kb deletion, 4.2 kb deletion and 20.5 kb deletion resulted in PCR products of 2,029 bp, 1,628 bp, and 1,007 bp, respectively.

#### Screening for a+ and a^0^ α-thalassemia caused by non-deletion mutations.

The three exons of each alpha 2 and alpha 1 gene were amplified and sequenced to determine all non-deletional mutations that cause a^+^ and a^0^ α-thalassemia. Each 25 uL reaction contained 0.5 μM of each primer hem alpha F, hem alpha 1 R, hem alpha 2 R, and also 1× Q-solution 2.5 U HotStarTaq DNA polymerase in supplied reaction buffer (Qiagen) and 100 ng of genomic DNA. Reactions were carried out on a thermal cycler (Gene Amp 2700, Applied Biosystems), with an initial 15-minute denaturation at 95°C, 38 cycles of 95°C for 20 seconds, 60°C for 20 seconds, 72°C for 90 seconds, and a final extension at 72°C for 5 minutes. Following amplification, 5 μL of product was electrophoresed through a 1.5% agarose gel with 0.6 μg/mL ethidium bromide in 1× TBE buffer at 7 volts/cm for 1 hour. The gel was visualized on an UV transilluminator. Resulting PCR products were purified with a QIAquick PCR Purification Kit (Qiagen) and sequenced on both strands with the Big Dye Terminator v3.1 sequencing kit on an ABI 3130XL sequencer (Applied Biosystems). The Sequence reads were aligned against reference genes for hemoglobin alpha 1 and 2 (NCBI Gene ID: 3039 and 3040, GRCh37.p10) and polymorphisms were identified according to human dbSNP (Build 137). Sequence analysis was done by the Geneious 6.1.5 software package.

All volunteers gave written informed consent before screening and being enrolled in the study. The trial was performed in accordance with Good Clinical Practices, an Investigational New Drug (IND) application filed with the U.S. Food and Drug Administration (US FDA) (IND 14267), and an Investigational Medical Product Dossier (IMPD) filed with the Tanzanian Food and Drug Administration (TFDA). The protocol was approved by institutional review boards (IRBs) of the Ifakara Health Institute ((IHI/IRB/No25) and National Institute for Medical Research Tanzania (NIMR/HQ/R.8a/Vol.IX/1217), and the Ethikkommission beider Basel (EKBB), Basel, Switzerland (EKBB 319/11). The protocol was also approved by TFDA (Ref. No. CE.57/180/04A/50), and the trial was registered at ClinicalTrials.gov (NCT01540903).

### Intervention and randomization.

The intervention material was aseptic, purified, cryopreserved PfSPZ (PfSPZ Challenge) isolated from salivary glands of aseptically reared mosquitoes.^16^,^17^ Details regarding the production, cryopreservation quality control, potency, and CHMI have been described.^11^–^13^,^18^,^19^ The lot of PfSPZ Challenge used in this study had been manufactured and then cryopreserved in liquid nitrogen vapor phase (LNVP) 12 months before administration. It was a different lot than the lot used in the Netherlands^12^; however, it was the same lot that was used in Oxford in which 5 of 6 volunteers who received 2,500 PfSPZ ID and 6 of 6 volunteers who received 25,000 PfSPZ IM developed malaria.^13^ The results of the sporozoite membrane integrity (viability) and 6-day hepatocyte potency assay (potency) were similar to those for the previous trials (Supplemental Table 1).^12^,^13^

Thirty eligible volunteers were randomly allocated to the experimental (PfSPZ Challenge) or control (normal saline) groups. Twenty-four volunteers received PfSPZ Challenge and six received normal saline (BD Medical/Surgical, BD Pre-filled Normal Saline Flush Syringe, Columbus, Nebraska). The volunteers and clinicians were blinded as to whether the volunteers received PfSPZ Challenge or normal saline.

### CHMI.

#### Administration of PfSPZ Challenge and immediate follow-up.

PfSPZ Challenge was administered ID in two different dose groups. Twelve volunteers were inoculated with 10,000 PfSPZ ID in two injection sites, one 50 μL injection in each deltoid, each injection containing 5,000 PfSPZ. Twelve volunteers were inoculated with 25,000 PfSPZ ID in four injection sites, two 10 μL injections in each deltoid, each injection containing 6,250 PfSPZ. Three control volunteers were assigned to each of the two dose groups and were inoculated with normal saline in the same way as those in that experimental group.

Immediately before use, a vial of PfSPZ Challenge was thawed and diluted with phosphate buffered saline containing human serum albumin in an aseptic environment. It was then injected by a blinded nurse within 30 minutes of thawing. After injection, volunteers were observed in the injection room for at least 5 minutes and thereafter were escorted by a nurse to the ward.

#### Diagnosis of malaria.

Thick blood smears were obtained every 12 hours on Days 5 through 14 after injection with saline or PfSPZ Challenge and daily on Days 15 through 21 until positive or until Day 21. After initiation of treatment of a positive thick smear, thick smears were assessed until three consecutive daily smears were negative after treatment. Thick smears were also assessed on Day 28 after CHMI.

Slide preparation and reading was performed following a standard procedure. In brief, 10 μL of blood was placed uniformly on a 10 mm × 20 mm area of the slide, air dried, and stained with Giemsa, pH 7.2. Microscopes were calibrated and the number of passes/fields required to read 0.5 μL blood was determined. This amount of blood was assessed for the primary reads to determine if the slide was negative. If the volunteer was symptomatic, double this amount of blood was read. A blood smear was declared positive when one reader saw two parasites in 0.5 μL of blood and the presence of parasites was independently confirmed by a second reader. The pre-patent period was defined as the period between inoculation of PfSPZ Challenge and appearance of the first positive blood smear. Retrospectively, parasitemias were also determined by quantitative polymerase chain reaction (qPCR) performed on all samples collected after CHMI, as previously described.^20^

#### Treatment of malaria.

Those who became smear positive were treated with a standard 3-day regimen of artemether/lumefantrine (Coartem), and were discharged after three consecutive negative smear results. Those who did not become positive during the first 21 days after CHMI were discharged and returned on Day 28. On this day, the study was unblinded and those who had received PfSPZ Challenge and had not developed infection were treated with artemether/lumefantrine (Coartem) irrespective of the blood smear results. All volunteers were seen on Days 56 and 168.

#### Assessment of adverse events.

The volunteers were observed in the ward for 24 hours after administration of PfSPZ Challenge and discharged to home. They were given diaries and thermometers for recording of adverse events and temperatures. Volunteers returned on Day 5 after administration of PfSPZ Challenge for admission to the ward for assessment of safety and diagnosis and treatment of malaria.

During the period of follow-up all symptoms and signs (solicited and unsolicited) were recorded and graded by the attending physician as follows: mild (easily tolerated), moderate (interferes with normal activity), or severe (prevents normal activity). Axillary temperature was recorded as grade 1 (> 37.5–38.0°C), grade 2 (> 38.0–39.0°C), or grade 3 (> 39.0°C). Hematological and biochemical parameters were assessed minimally on Days 5, 9, 12, 28, 56, and 168 after inoculation of PfSPZ Challenge, and on the day of parasite positivity (day of initiating treatment). For those individuals who did not become positive by thick blood smear, these assays were also conducted on Days 15, 18, and 21 after inoculation of PfSPZ Challenge. Results were graded according to a predetermined table (Supplemental Table 2 adapted from FDA guidelines, http://www.fda.gov/downloads/BiologicsBloodVaccines/GuidanceComplianceRegulatoryInformation/Guidances/Vaccines/ucm091977.pdf). Adverse events were divided into those that occurred during the 5 days after inoculation of PfSPZ Challenge, and were attributed to the administration of the study product, and those that occurred from Day 6 onward, and were attributed to Pf infection (malaria).

The possibility of cardiac damage was assessed, because a cardiac-related serious adverse event (SAE) had been reported in 2007 in the Netherlands in a volunteer who was immunized with an experimental Pf subunit vaccine, underwent CHMI by exposure to the bites of five Pf-infected mosquitoes, developed malaria, and was treated with an antimalarial.^21^ In case symptoms or signs that could be related to a cardiac event developed during the study, blood was collected at baseline to be able to determine if there was any difference in the results of assays used to assess cardiac damage (e.g., troponins) before the trial began and at the time of such an event. We note that after our clinical trial in Tanzania, another cardiac event occurred in a volunteer in a PfSPZ-CVac vaccine trial in the Netherlands after CHMI by mosquito bite, diagnosis of malaria, and initiation of treatment.^22^

#### Genotyping of parasites.

At the time of diagnosis and before treatment 4 mL of blood were collected for genotyping by microsatellite analysis to determine if the parasites were derived from PfSPZ Challenge (Pf NF54) or from a naturally acquired infection. The DNA was isolated from blood specimens using the QIAamp DNA Blood Midi Kit (Qiagen). Microsatellite markers Poly alpha, PfPK2, TA81, ARA2, TA87, and TA40 were amplified using hemi-nested PCR.^23^,^24^ Capillary electrophoresis was performed using an Applied Biosystems 3730XL 96-capillary DNA sequencer and software. Capillary electrophoresis output files were analyzed using Genemapper 4.0 (Applied Biosystems). Genomic control strains 3D7 and HB3 (ATCC-MR4, Manassas VA) were included to determine characteristic peak morphology for each microsatellite locus and control for slight variations among runs. Microsatellite peaks above the threshold of 100 relative fluorescent units exhibiting characteristic morphology were scored. Peak sizes were determined by manual inspection of each electropherogram and then normalized against the Pf 3D7 control. Normalized peak sizes were compared with those observed in Pf NF54.

### Statistical analysis.

Data analysis was performed using SAS software version 9.3 (SAS Institute Inc., Cary, NC). Descriptive statistics were assessed, specifically the geometric mean, for the parasitemia results. Thick blood smear and qPCR results were compared between the 10,000 and 25,000 PfSPZ groups using a non-parametric test (Wilcoxon rank-sum test, two-tailed). The effects of α-thalassemia heterozygosity on parasitemia were compared within each group by non-parametric tests (Wilcoxon rank-sum test, two-tailed). Proportions were compared using χ^2^ test (two-tailed).

## Results

### Volunteers.

**Figure 1.**
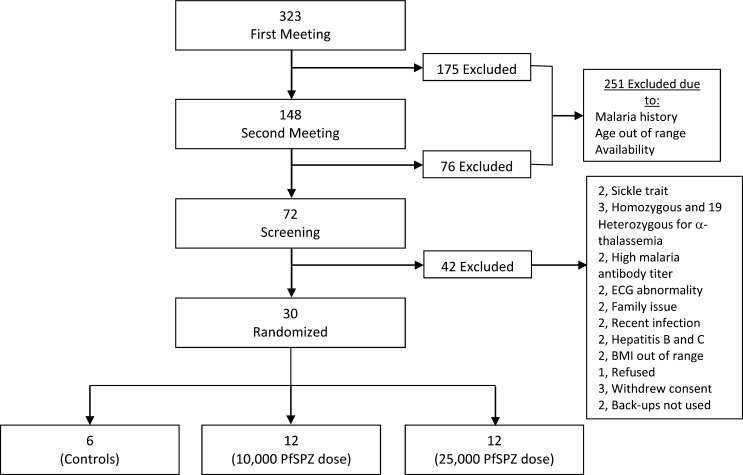
Flow chart of recruitment and study design.

In total 323 volunteers were recruited from higher learning institutions during the first information meeting. Out of these, 30 met eligibility criteria and were enrolled and randomized to the 10,000 PfSPZ dose (*N* = 12), 25,000 PfSPZ dose (*N* = 12), or control (*N* = 6) groups (Figure 1 and Table 1). In groups 1, 2, and the control group, 7, 1, and 2 volunteers were heterozygous for the α-thalassemia trait, respectively.

### Parasitemia.

#### Thick blood smear.

Eleven volunteers who received 10,000 PfSPZ (Table 2A) and 10 volunteers who received 25,000 PfSPZ (Table 2B) developed parasitemia detected by thick blood smear and confirmed by qPCR. One of the other two subjects in the 25,000 PfSPZ group was treated for a false positive thick blood smear on Day 11 (qPCR was negative), and was eliminated from the analysis. The second other volunteer in the 25,000 PfSPZ group was treated for a false positive thick blood smear on Day 19; qPCR was negative throughout for this volunteer, and this volunteer was considered to have not developed parasitemia as no subject in the study developed parasitemia by qPCR after Day 16, and this volunteer was followed by qPCR through Day 19. Thus, 11 of 12 volunteers in the 10,000 PfSPZ group and 10 of 11 evaluable volunteers in the 25,000 PfSPZ group developed Pf parasitemia after ID injection of PfSPZ Challenge. Volunteers in the 10,000 PfSPZ group had a significantly different pre-patent period than in the 25,000 PfSPZ group (geometric mean [GM] of 15.4 and 13.5 days, Wilcoxon, *P* = 0.023). The GM parasite densities were 8.9 and 7.0 parasites/μL blood, respectively, at the time of first thick smear positivity.

#### qPCR.

**Figure 2.**
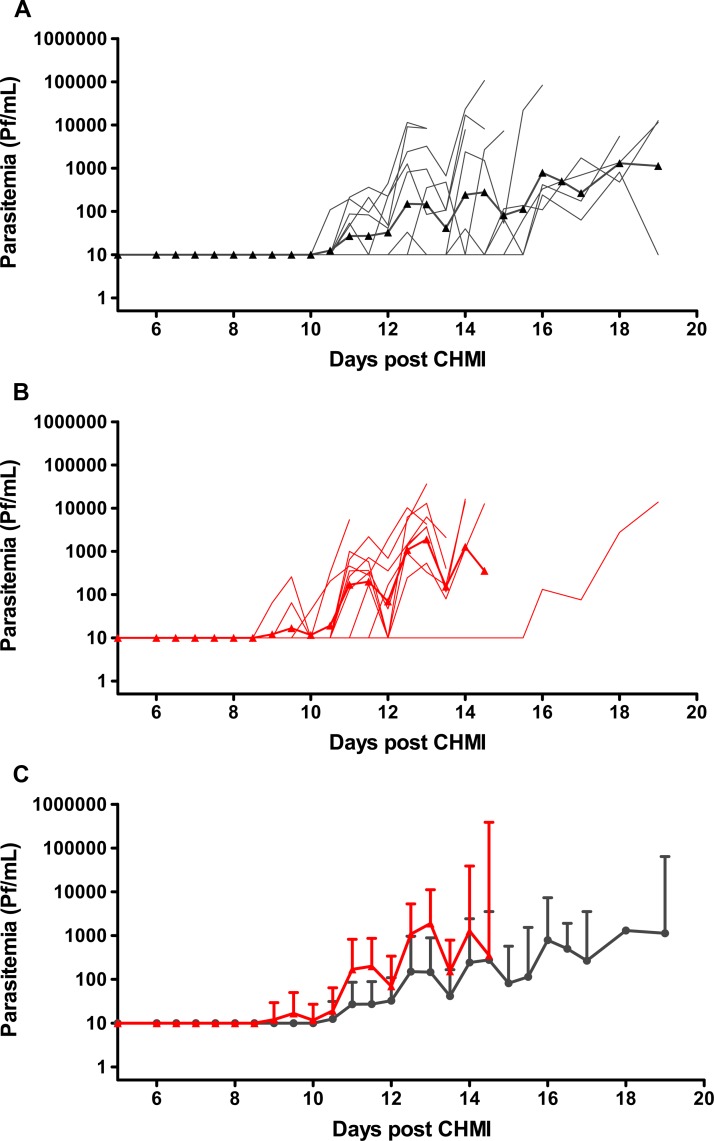
Parasite density as measured by qPCR in the 10,000 (**A**) and 25,000 (**B**) PfSPZ dose groups. Panels A (*N* = 11) and B (*N* = 10) show individual and geometric mean parasite density (parasites/mL) of positive volunteers from day of inoculation through last day of positivity after initiation of treatment. Panel C shows an overlay of geometric mean parasite densities with confidence intervals (95%) of positive volunteers in each group (Black line = Group 1, 10,000 PfSPZ; Grey line = Group 2, 25,000 PfSPZ).* *For Panel B, the geometric mean was calculated excluding the single volunteer who first became positive on Day 16.

Parasitemia was determined by qPCR on all samples collected after CHMI. The qPCR was performed retrospectively, after volunteers had been diagnosed and treated. The sensitivity of qPCR was considered to be 20 parasites/mL of blood. The qPCRs were first positive 9.0 to 16.0 days after inoculation of PfSPZ Challenge (Table 2). Consistent with thick blood smear results, the volunteers in the 10,000 PfSPZ group had a longer time to positive qPCR than those in the 25,000 PfSPZ group (GM of 12.6 and 11.1 days, respectively), however the difference did not reach the level of statistical significance (Wilcoxon, *P* = 0.076). The GM parasite densities were 0.11 and 0.16 parasites/μL blood (110 and 160 parasites/mL blood), respectively, at the time of first qPCR positivity. The GM parasite densities by qPCR at the time of thick smear diagnosis in the two groups were 4.1 and 1.6 parasites/μL, respectively. qPCR was negative throughout the 21-day follow-up for the slide-negative volunteer who received 10,000 PfSPZ, and through Day 19 for the subject who received 25,000 PfSPZ and was treated on Day 19. It was also negative in all normal saline controls, except for one specimen on Day 19, which was determined to be caused by mislabeling of a specimen from a PfSPZ Challenge subject. Parasite growth was cyclical and similar in both dose groups (Figure 2). Using a previously described method,^25^ the parasite multiplication rate in the bloodstream could not be determined with confidence because of the high variability of the amplification dynamics among individual subjects. However, there did not appear to be a significant difference between the replication rate in the Tanzanians and the replication rate in the Dutch subjects.^12^

#### Parasite genotypes.

Parasites from the positive volunteers were genotyped at six polymorphic microsatellite loci to confirm that they were from PfSPZ Challenge (Pf NF54) and not acquired by natural exposure to infected mosquitoes in the Bagamoyo area. Parasites from all 21 volunteers had identical microsatellite signatures to Pf NF54 (Table 3), indicating that they were infected with the challenge strain and not locally transmitted Pf parasites. Later, on Days 67 and 89 after CHMI, two volunteers developed Pf malaria. These infections were also genotyped and both carried non-Pf NF54 alleles at all six microsatellite loci (Table 3), indicating that they were infected with naturally acquired, locally transmitted Pf parasites.

#### Effect of α-thalassemia heterozygosity on parasitemia.

The GM parasite density for volunteers who were heterozygous for α-thalassemia and those who were non-α-thalassemic in the 10,000 PfSPZ were not significantly different at the time of detection of parasites by microscopy, 11.1 versus 6.9 Pf/μL blood, respectively (Wilcoxon, *P* = 0.5498) (Table 4). The GM time to blood smear positivity (pre-patent period) was also not significantly different between α-thalassemia heterozygous and non- α-thalassemia volunteers, 15.4 and 15.4 days, respectively, in the 10,000 PfSPZ group (Wilcoxon, *P* = 0.7922). In the 25,000 PfSPZ group, the parasite densities at the time of detection of parasites by microscopy was 4.0 parasites/μL blood in the one α-thalassemia heterozygous volunteer and 7.4 parasites/μL blood (GM) in non-α-thalassemia volunteers. The time to blood slide positivity in the single α-thalassemia heterozygous subject was no different from GM time to blood slide positivity in the non-α-thalassemia subjects, 12.7 versus 13.6 days (Table 4).

### Adverse events (AEs).

#### Clinical adverse events.

A summary of the number of volunteers in each group with AEs during the 28 days after injection of normal saline or PfSPZ Challenge is shown in Table 5. The occurrence of AEs was similar in all groups. Most AEs recorded after administration of PfSPZ were consistent with symptoms and/or signs associated with clinical malaria. Table 6 provides the total number of AEs that occurred during Days 0–28 broken down by Grade (1–3); 75% were Grade 1. Table 7 delineates the specific solicited and unsolicited AEs that occurred during Days 0–28 after injection of normal saline or PfSPZ Challenge. There were 63 AEs in groups 2 (10,000 PfSPZ) and 3 (25,000 PfSPZ) and only nine of the 63 AEs (14%) occurred during the first 5 days after injection of PfSPZ Challenge. There were no acute systemic allergic reactions after the injection. The most common study-related events were headache, malaise, fatigue, and arthralgia, all symptoms consistent with malaria. Surprisingly, saline control subjects who did not have malaria had these symptoms at a frequency similar to that of subjects who received PfSPZ Challenge.

#### Clinical serious adverse events *(*SAEs*)*—all unrelated.

There were two subjects who had unrelated SAEs in this study. The first SAE was in a 26-year-old volunteer who was diagnosed as having gastroenteritis/dysentery of grade 3 severity at the time of hospitalization, which was 33 days post administration of PfSPZ Challenge. The second SAE occurred in a 22-year-old volunteer who was admitted to the hospital and diagnosed with severe malaria (axillary temperature > 40°C, altered mental status, and smear positive) on day 89 after administration of PfSPZ Challenge (the volunteer was previously treated and successfully cleared of parasites on Day 14 post inoculation of PfSPZ Challenge). Parasites taken from this volunteer at the time of diagnosis of severe malaria were genotyped using microsatellite markers, and these parasites were shown not to be the Pf NF54 parasites in PfSPZ Challenge (see above, and Table 3). Both volunteers with SAEs recovered uneventfully.

#### Laboratory test abnormalities.

Serum chemistries (glucose, creatinine, aspartate aminotransferase [AST], alanine aminotransferase [ALT], and bilirubin) and hematological parameters (e.g., hemoglobin, white blood cell count, platelet count) were assessed beginning on Day 5 after inoculation of normal saline or PfSPZ Challenge. When assessed on Day 5 after inoculation of normal saline and PfSPZ Challenge, laboratory abnormalities were infrequent and self-limited and similar in incidence rate among all three groups (Supplemental Table 3). From Day 6 to Day 28 post inoculation the incidence rate of abnormalities was slightly increased, but the incidence rate of abnormalities was similar in normal saline controls and those who received PfSPZ Challenge (Supplemental Table 3).

## Discussion

In this study, we showed for the first time that inoculation of healthy, young adult, African males with aseptic, purified, cryopreserved *P. falciparum* sporozoites, a product called PfSPZ Challenge, was safe, well tolerated, and infective. In fact, the infection rates in the Tanzanians were as good, if not better than the infection rates in young adult Dutch with no previous exposure to malaria.^12^

Eleven of 12 subjects who received 10,000 PfSPZ ID in two divided 50 μL doses and 10 of 11 who received 25,000 PfSPZ ID in four divided 10 μL doses became parasitemic. In the Netherlands, 5 of 6 who received 10,000 and 5 of 6 who received 25,000 PfSPZ ID in two divided 50 μL doses became parasitemic.

However, the GM pre-patent period for the Tanzanians who received the 10,000 PfSPZ dosage regimen was 15.4 days, and the GM pre-patent period for the Dutch was 12.6 days (*P* = 0.0192, Wilcoxon 2-tailed). There are multiple possible explanations for the prolonged pre-patent period in the Tanzanians. One is that naturally acquired immunity reduced the number of parasites that invaded or fully developed in the liver, thereby reducing the numbers of parasites that were released from the liver and prolonging the pre-patent period. Another possibility is that the replication rate in the blood was reduced as a result of naturally acquired immunity or innate resistance, and that this reduced replication prolonged the pre-patent period. Unfortunately, the variability in the qPCR results did not allow for definitively determining if this was the case, but it appeared that the replication rate was similar to the 11.5-fold replication rate every 48 hours seen in the Dutch volunteers.^12^ Thus, we do not know why the pre-patent period was longer in the Tanzanians. However, because the replication rates seemed to be similar in the Dutch and Tanzanian subjects, we think that there may have been fewer merozoites released from the livers of the Tanzanians.

Interestingly, the GM pre-patent period in the group that received 25,000 PfSPZ was significantly different (13.5 days) than was the pre-patent period in the group that received 10,000 PfSPZ (15.4 days) (*P* = 0.023). This 2-day difference can only partially be accounted for by increasing the numbers of PfSPZ by 2.5-fold. It is likely that more efficient administration of the PfSPZ contributed to the delay by increasing the number of sites of administration from two to four and decreasing the volume of the injections from 50 to 10 μL, as we have seen in mice.^14^

Hemoglobinopathies, disorders of hemoglobin structure, and production, are one of the most common monogenic disorders in humans. α-thalassemia is a hemoglobinopathy resulting from deletion of one (−α) or both (–) α genes from chromosome 16. Its wide distribution in populations living in places like Tanzania, where malaria has been or still is present, has been hypothesized to result from protection against severe or lethal malaria.^26^ This study was not designed (i.e. powered) to be able to assess the differences in infection rates, pre-patent periods, and parasite densities between individuals with and without α-thalassemia trait. Nonetheless, there was no indication of any differences between those carrying this trait and those who did not have it. This finding is consistent with a number of field studies that showed that individuals who were heterozygous or homozygous for α-thalassemia had similar rates of asymptomatic parasitemia and similar manifestations of uncomplicated malaria as non-thalassemic individuals.^27^

Administration of PfSPZ Challenge was extremely well tolerated. There were no serious adverse events. In fact, the incidence rate of AEs was the same in the control group that received normal saline as it was in the groups that received PfSPZ Challenge (Tables 5–7). Very few of the AEs occurred during the first 5 days after inoculation of PfSPZ Challenge, and thus very few were attributed to the injection of PfSPZ Challenge (Table 7). Most occurred during the period when the volunteers were being diagnosed with Pf parasitemia and being treated. However, even during this period the incidence rate was similar in the control and experimental groups. One of the striking findings was how few symptoms and signs attributable to Pf parasitemia were experienced by the subjects who developed malaria. In the Dutch study 9 of 18 volunteers reported fever, whereas only 2 of 24 (*P* = 0.004, Fisher's exact test, 2-tailed) reported fever in this study. In the Netherlands 5 of 18 had chills, whereas in this study only 1 of 24 had chills (*P* = 0.068).^12^ We assume this low level of symptoms and signs was attributable to naturally acquired or innate immunity, which ameliorated clinical manifestations.

A panel of six polymorphic microsatellite loci was successfully used to identify challenge strain (Pf NF54) infections and differentiate them from naturally acquired *P. falciparum* parasites circulating in Bagamoyo. This technique has been previously shown to be highly effective at differentiating genetic variants of *P. falciparum* within countries and across larger geographic regions.^28^–^32^ Here, we show that the assay is also sensitive enough to generate reliable multi-locus genotypes from infected blood with parasite densities as low as five parasites/μL blood. Although microsatellites are useful in identifying the infecting strain, they do not provide information on genetic regions of vaccine-induced selection or escape following heterologous challenge. Future studies will include genome-wide characterization of breakthrough infections in PfSPZ Vaccine^11^,^18^,^19^ trials using DNA microarrays and genome sequencing to assess cross-strain protection and identify loci under vaccine-induced immune selection. This approach may help to pinpoint the genetic regions encoding the key antigens responsible for driving strain-specific immune responses and inform the development of next-generation multivalent whole organism vaccines in the event that efficacy is found to be strain-specific.

In conclusion, we have established the foundation for using CHMI with PfSPZ Challenge in Africans to establish the efficacy of new interventions against malaria and to study the mechanisms of protection conferred by hemoglobinopathies, glucose 6 phosphate dehydrogenase deficiencies, and innate and acquired immunity to malaria in settings where malaria is endemic. Improvements in administration, such as direct venous inoculation (DVI) (Mordmueller B, submitted), will soon be used in Bagamoyo in CHMI studies to assess the protective efficacy of the PfSPZ Vaccine^19^ in Tanzanians.

## Supplementary Material

Supplemental Tables.

## Figures and Tables

**Table 1:** Volunteer characteristics*

	Control (normal saline)	Group 1 (10,000 PfSPZ)	Group 2 (25,000 PfSPZ)
	*N* = 6	*N* = 12	*N* = 12
Sex			
Male	6	12	12
Age at screening (years)			
Mean ± SD	25.7 ± 3.0	25.9 ± 1.6	24.6 ± 2.3
Median	25.2	25.5	24.5
Min, max	21.5, 30.9	24.3, 30.5	20.7, 27.2
BMI (kg/m^2^)			
Mean ± SD	20.7 ± 2.4	21.7 ± 3	20.5 ± 2.3
Median	19.3	20.7	19.7
Min, max	19, 24	18.4, 27.9	17.3, 25.4
Height (cm)			
Mean ± SD	168 ± 4.3	168.5 ± 8.2	171.7 ± 5.6
Median	166	169	172
Min, max	164, 174	158, 182	162, 179
Weight (kg)			
Mean ± SD	58.4 ± 6.3	61.7 ± 9.8	60.5 ± 8.8
Median	58.3	60.8	60.5
Min, max	51, 66	47, 79	45.5, 81.5

* BMI = body mass index.

**Table 2:** A. Thick smear and qPCR results, group 1 (10,000 PfSPZ)

Volunteer code	Thick smear		qPCR		
	Pre-patent period (day)	Parasite density at diagnosis (Pf/μL)	qPCR positive (day)	Parasite density at first day positive (Pf/μL)	Parasite density by qPCR at time of diagnosis by thick smear (Pf/μL)
10002-20	18.6	5.0	16.0	0.24	0.01
10023-20	18.7	15.0	15.0	0.12	13.00
30035-20	18.7	5.0	14.0	0.04	11.00
40010-20	N/A	N/A	N/A	N/A	N/A
50041-20	14.6	4.0	13.0	0.36	3.00
60008-20	12.8	4.0	10.5	0.11	8.00
60026-20	12.7	11.0	11.5	0.21	9.00
70001-20	14.2	80.0	11.0	0.21	23.00
70014-20	15.8	21.0	12.5	0.03	83.00
70031-20	14.2	11.0	11.0	0.05	17.00
70044-20	17.6	6.0	15.0	0.07	6.00
90047-20	13.7	4.0	11.0	0.09	0.10
Geom. mean	15.4	8.9	12.6	0.11	4.10
No. of positives	11/12		11/12		
B. Thick smear and qPCR results, group 2 (25,000 PfSPZ)					
20056-20	18.7	5	16.0	0.13	14.00
20064-20	11.1	7	9.0	0.07	5.00
20070-20	12.6	13	9.5	0.07	5.00
30053-20	13.7	4	12.0	0.17	0.17
30060-20	13.5	7	11.0	1.00	0.40
40055-20	N/A	N/A*	N/A	N/A	N/A
40068-20	13.4	7	11.0	0.26	2.00
50050-20	12.7	7	10.0	0.04	10.00
50057-20	N/A	N/A†	N/A	N/A	N/A
60051-20	12.7	4	11.0	0.13	1.00
60072-20	14.0	7	11.5	0.17	1.00
80058-20	13.7	15	11.0	0.36	0.12
Geom. mean	13.5	7	11.1	0.16	1.60
No. of positives	10/11		10/11		

* This volunteer was treated on Day 19 as a result of reporting of a positive thick blood smear. Review of this thick blood smear indicated that it was negative, and all qPCR results on this volunteer were negative. Thus, this volunteer was considered as negative for *Plasmodium falciparum* infection for subsequent analyses, because no volunteers first became qPCR positive on Day 19 or later.

† This volunteer was treated on Day 11 as a result of reporting of a positive thick blood smear. Review of this thick blood smear indicated it was negative, and all qPCR results on this volunteer were negative. Thus, it is not known if this volunteer would have developed *Plasmodium falciparum* infection, and this volunteer has been excluded from the analysis. Thus, 11 volunteers were considered to be in this group, and 10 were documented to have developed *P. falciparum* parasitemia.

**Table 3:**
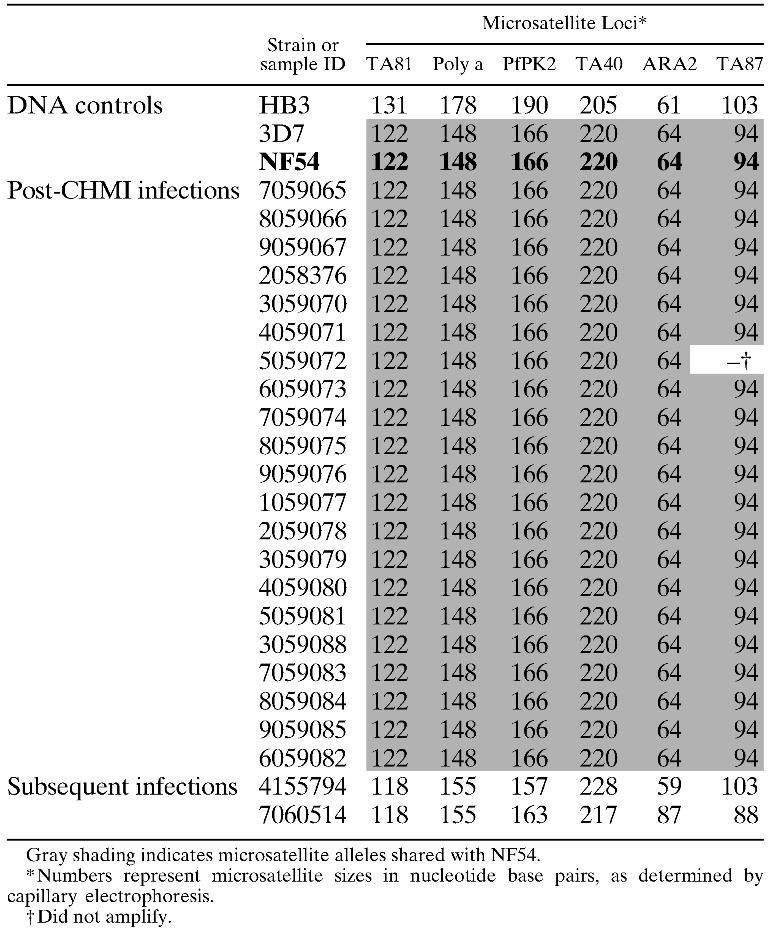
Microsatellite genotypes of Pf infections in volunteers after PfSPZ Challenge

**Table 4:** Pre-patent periods and parasite densities by microscopy by α-thalassemia status

Heterozygous			Normal		
Volunteer ID	Pre-patent period (Days)	Parasite density at diagnosis	Volunteer ID	Pre-patent period (Days)	Parasite density at diagnosis
Group 1*					
90047-20	13.74	4.0	50041-20	14.64	4.0
70001-20	14.11	80.0	60008-20	12.75	4.0
60026-20	12.71	11.0	70044-20	17.63	6.0
70014-20	15.76	21.0	70031-20	14.16	11.0
10002-20	18.64	5.0	10023-20	18.73	15.0
30035-20	18.66	5.0			
Geom. mean	15.43	11.1		15.42	6.9
No. positive	6/7			5/5	

Group 2*					
60051-20	12.72	4.0	30053-20	13.72	4.0
			80058-20	13.68	15.0
			20056-20	18.67	5.0
			40068-20	13.44	7.0
			30060-20	13.47	7.0
			50050-20	12.72	7.0
			60072-20	14.04	7.0
			20070-20	12.62	13.0
			20064-20	11.05	7.0
Geom. mean	12.72	4.0		13.59	7.4
No. positive	1/1			9/10	

* The proportion of volunteers who were heterozygous for α-thalassemia trait was higher in the 10,000 PfSPZ group than in the 25,000 PfSPZ group (chi-squared, 2-tailed, *P* = 0.011). There were no statistically significant differences in infection rates, parasitemia, or parasites/μL blood between subjects heterozygous for α-thalassemia trait, and those who were not.

**Table 5:** Number of volunteers with clinical adverse events, Days 0–28 post injection*

	Saline control (*N* = 6)	Group 1 (10,000 PfSPZ) (*N* = 12)	Group 2 (25,000 PfSPZ) (*N* = 12)
Adverse events (no. of volunteers, %)	n (%)	n (%)	n (%)
Any adverse event	6 (100)	9 (75)	9 (75)
Serious adverse event	0	0	0
Solicited adverse event	6 (100)	9 (75)	9 (75)
Unsolicited adverse event	2 (33)	6 (50)	2 (17)

* A volunteer was counted at most once within each event type.

**Table 6:** Number of clinical adverse events, Days 0–28 post-injection

	Saline control (*N* = 6)	Group 1 (10,000 PfSPZ) (*N* = 12)	Group 2 (25,000 PfSPZ) (*N* = 12)
Total number of AEs	22	42	42
Total solicited AEs			
Grade 1	14	30	34
Grade 2	4	2	6
Grade 3	2*	1†	0
Total unsolicited AEs			
Grade 1	2	8	1
Grade 2	0	0	1
Grade 3	0	1‡	0
Total SAEs	0	0	0

An adverse event (AE) was recorded more than once if it resolved and subsequently reappeared during the 28-day interval.

* Severe rash and pruritis (each grade 3) in a volunteer beginning 10 days post-injection.

† Severe headache attributable to controlled human malaria infection (CHMI).

‡ Pharyngitis, etiology unclear, resolved after 3 days.

SAE = serious adverse event.

**Table 7:** Specific solicited and unsolicited adverse events, Days 0–28 post–injection*

Adverse event	Saline control	Group 1 10,000 PfSPZ	Group 2 25,000 PfSPZ
	*N* = 6	*N* = 12	*N* = 12
Total AEs	21 (2)	30 (3)	34 (6)
Fever	0	0	2
Headache	6 (1)	7	7 (1)
Malaise	1	5 (1)	4
Fatigue	3 (1)	5	6 (2)
Myalgia	3	2	1
Arthralgia	3	2	4
Nausea and/or vomiting	0	0	0
Chills	0	0	1
Diarrhea	0	0	0
Constipation	0	1	0
Abdominal pain	1	0	1 (1)
Chest pain/discomfort	0	1	0
Palpitations	0	0	0
Shortness of breath	0	0	0
Dizziness	1	0	0
Erythema, swelling, or pruritis at injection site	0	0	3 (2)
Rash or pruritus remote to injection site	2	0	0
Itching throat	0	0	1
Loss of appetite	0	3 (1)	1
Pain on swallowing/pharyngitis	1	2	0
Back pain	0	1 (1)	0
Neck pain	0	1	0
Elevated axillary temp.	0	0	3

* A volunteer was counted at most once within each event type, even if the AE cleared and reappeared. All AEs were considered either possibly or probably related to the injection of study product or malaria. Numbers in parentheses occurred within the first 5 days after injection of study product.

## References

[1] Mayne B (1933). The injection of mosquito sporozoites in malaria therapy. Public Health Rep.

[2] Mayne B, Young M (1941). The technique of induced malaria as used in the South Carolina State Hospital. J Vener Dis Inf.

[3] Boyd MF (1949). Malariology: A Comprehensive Survey of All Aspects of This Group of Diseases from a Global Standpoint.

[4] Ifediba T, Vanderberg JP (1981). Complete *in vitro* maturation of *Plasmodium falciparum* gametocytes. Nature.

[5] Campbell CC, Collins WE, Nguyen Dinh P, Barber A, Broderson JR (1982). *Plasmodium falciparum* gametocytes from culture *in vitro* develop to sporozoites that are infectious to primates. Science.

[6] Chulay JD, Schneider I, Cosgriff TM, Hoffman SL, Ballou WR, Quakyi IA, Carter R, Trosper JH, Hockmeyer WT (1986). Malaria transmitted to humans by mosquitoes infected from cultured *Plasmodium falciparum*. Am J Trop Med Hyg.

[7] Church LW, Le TP, Bryan JP, Gordon DM, Edelman R, Fries L, Davis JR, Herrington DA, Clyde DF, Shmuklarsky MJ, Schneider I, McGovern TW, Chulay JD, Ballou WR, Hoffman SL (1997). Clinical manifestations of *Plasmodium falciparum* malaria experimentally induced by mosquito challenge. J Infect Dis.

[8] Epstein JE, Rao S, Williams F, Freilich D, Luke T, Sedegah M, de la Vega P, Sacci J, Richie TL, Hoffman SL (2007). Safety and clinical outcome of experimental challenge of human volunteers with *Plasmodium falciparum*-infected mosquitoes: an update. J Infect Dis.

[9] Roestenberg M, O'Hara GA, Duncan CJ, Epstein JE, Edwards NJ, Scholzen A, van der Ven AJ, Hermsen CC, Hill AV, Sauerwein RW (2012). Comparison of clinical and parasitological data from controlled human malaria infection trials. PLoS ONE.

[10] Laurens MB, Duncan CJ, Epstein JE, Hill AV, Komisar JL, Lyke KE, Ockenhouse CF, Richie TL, Roestenberg M, Sauerwein RW, Spring MD, Talley AK, Moorthy VS (2012). A consultation on the optimization of controlled human malaria infection by mosquito bite for evaluation of candidate malaria vaccines. Vaccine.

[11] Hoffman SL, Billingsley P, James E, Richman A, Loyevsky M, Li T, Charkravarty S, Gunasekera A, Chattopadhyay R, Li M, Stafford R, Ahumada A, Epstein JE, Sedegah M, Reyes S, Richie TL, Lyke KE, Edelman R, Laurens MB, Plowe CV, Sim BKL (2010). Development of a metabolically active, non-replicating sporozoite vaccine to prevent *Plasmodium falciparum* malaria. Hum Vaccin.

[12] Roestenberg M, Bijker EM, Sim BK, Billingsley PF, James ER, Bastiaens GJ, Teirlinck AC, Scholzen A, Teelen K, Arens T, van der Ven AJ, Gunasekera A, Chakravarty S, Velmurugan S, Hermsen CC, Sauerwein RW, Hoffman SL (2013). Controlled human malaria infections by intradermal injection of cryopreserved *Plasmodium falciparum* sporozoites. Am J Trop Med Hyg.

[13] Sheehy SH, Spencer AJ, Douglas AD, Sim BK, Longley RJ, Edwards NJ, Poulton ID, Kimani D, Williams AR, Anagnostou NA, Roberts R, Kerridge S, Voysey M, James ER, Billingsley PF, Gunasekera A, Lawrie AM, Hoffman SL, Hill AV (2013). Optimizing controlled human malaria infection studies using cryopreserved parasites administered by needle and syringe. PLoS ONE.

[14] Ploemen IH, Chakravarty S, van Gemert GJ, Annoura T, Khan SM, Janse CJ, Hermsen CC, Hoffman SL, Sauerwein RW (2013). *Plasmodium* liver load following parenteral sporozoite administration in rodents. Vaccine.

[15] Tan AS, Quah TC, Low PS, Chong SS (2001). A rapid and reliable 7-deletion multiplex polymerase chain reaction assay for alpha-thalassemia. Blood.

[16] Lyke KE, Laurens M, Adams M, Billingsley PF, Richman A, Loyevsky M, Chakravarty S, Plowe CV, Sim BK, Edelman R, Hoffman SL (2010). *Plasmodium falciparum* malaria challenge by the bite of aseptic *Anopheles stephensi* mosquitoes: results of a randomized infectivity trial. PLoS ONE.

[17] Laurens MB, Billingsley P, Richman A, Eappen AG, Adams M, Li T, Chakravarty S, Gunasekera A, Jacob CG, Sim BK, Edelman R, Plowe CV, Hoffman SL, Lyke KE (2013). Successful human infection with *P. falciparum* using three aseptic *Anopheles stephensi* mosquitoes: a new model for controlled human malaria infection. PLoS ONE.

[18] Epstein JE, Tewari K, Lyke KE, Sim BK, Billingsley PF, Laurens MB, Gunasekera A, Chakravarty S, James ER, Sedegah M, Richman A, Velmurugan S, Reyes S, Li M, Tucker K, Ahumada A, Ruben AJ, Li T, Stafford R, Eappen AG, Tamminga C, Bennett JW, Ockenhouse CF, Murphy JR, Komisar J, Thomas N, Loyevsky M, Birkett A, Plowe CV, Loucq C, Edelman R, Richie TL, Seder RA, Hoffman SL (2011). Live attenuated malaria vaccine designed to protect through hepatic CD8+T cell immunity. Science.

[19] Seder RA, Chang LJ, Enama ME, Zephir KL, Sarwar UN, Gordon IJ, Holman LA, James ER, Billingsley PF, Gunasekera A, Richman A, Chakravarty S, Manoj A, Velmurugan S, Li M, Ruben AJ, Li T, Eappen AG, Stafford RE, Plummer SH, Hendel CS, Novik L, Costner PJ, Mendoza FH, Saunders JG, Nason MC, Richardson JH, Murphy J, Davidson SA, Richie TL, Sedegah M, Sutamihardja A, Fahle GA, Lyke KE, Laurens MB, Roederer M, Tewari K, Epstein JE, Sim BK, Ledgerwood JE, Graham BS, Hoffman SL, for the VRC 312 Study Team (2013). Protection against malaria by intravenous immunization with a nonreplicating sporozoite vaccine. Science.

[20] Adegnika AA, Verweij JJ, Agnandji ST, Chai SK, Breitling LP, Ramharter M, Frolich M, Issifou S, Kremsner PG, Yazdanbakhsh M (2006). Microscopic and sub-microscopic *Plasmodium falciparum* infection, but not inflammation caused by infection, is associated with low birth weight. Am J Trop Med Hyg.

[21] Nieman AE, de Mast Q, Roestenberg M, Wiersma J, Pop G, Stalenhoef A, Druilhe P, Sauerwein R, van der Ven A (2009). Cardiac complication after experimental human malaria infection: a case report. Malar J.

[22] van Meer MP, Bastiaens GJ, Boulaksil M, de Mast Q, Gunasekera A, Hoffman SL, Pop G, van der Ven AJ, Sauerwein RW (2014). Idiopathic acute myocarditis during treatment for controlled human malaria infection: a case report. Malar J.

[23] Anderson TJ, Su XZ, Bockarie M, Lagog M, Day KP (1999). Twelve microsatellite markers for characterization of *Plasmodium falciparum* from finger-prick blood samples. Parasitology.

[24] Shaukat AM, Gilliams EA, Kenefic LJ, Laurens MB, Dzinjalamala FK, Nyirenda OM, Thesing PC, Jacob CG, Molyneux ME, Taylor TE, Plowe CV, Laufer MK (2012). Clinical manifestations of new versus recrudescent malaria infections following anti-malarial drug treatment. Malar J.

[25] Roestenberg M, de Vlas SJ, Nieman AE, Sauerwein RW, Hermsen CC (2012). Efficacy of preerythrocytic and blood-stage malaria vaccines can be assessed in small sporozoite challenge trials in human volunteers. J Infect Dis.

[26] Harteveld CL, Higgs DR (2010). Alpha-thalassaemia. Orphanet J Rare Dis.

[27] Taylor SM, Parobek CM, Fairhurst RM (2012). Hemoglobinopathies and the clinical epidemiology of malaria: a systematic review and meta-analysis. Lancet Infect Dis.

[28] Baliraine FN, Afrane YA, Amenya DA, Bonizzoni M, Vardo-Zalik AM, Menge DM, Githeko AK, Yan G (2010). A cohort study of *Plasmodium falciparum* infection dynamics in Western Kenya Highlands. BMC Infect Dis.

[29] Schultz L, Wapling J, Mueller I, Ntsuke PO, Senn N, Nale J, Kiniboro B, Buckee CO, Tavul L, Siba PM, Reeder JC, Barry AE (2010). Multilocus haplotypes reveal variable levels of diversity and population structure of *Plasmodium falciparum* in Papua New Guinea, a region of intense perennial transmission. Malar J.

[30] Griffing SM, Mixson-Hayden T, Sridaran S, Alam MT, McCollum AM, Cabezas C, Marquino Quezada W, Barnwell JW, De Oliveira AM, Lucas C, Arrospide N, Escalante AA, Bacon DJ, Udhayakumar V (2011). South American *Plasmodium falciparum* after the malaria eradication era: clonal population expansion and survival of the fittest hybrids. PLoS ONE.

[31] Mobegi VA, Loua KM, Ahouidi AD, Satoguina J, Nwakanma DC, Amambua-Ngwa A, Conway DJ (2012). Population genetic structure of *Plasmodium falciparum* across a region of diverse endemicity in West Africa. Malar J.

[32] Yalcindag E, Elguero E, Arnathau C, Durand P, Akiana J, Anderson TJ, Aubouy A, Balloux F, Besnard P, Bogreau H, Carnevale P, D'Alessandro U, Fontenille D, Gamboa D, Jombart T, Le Mire J, Leroy E, Maestre A, Mayxay M, Menard D, Musset L, Newton PN, Nkoghe D, Noya O, Ollomo B, Rogier C, Veron V, Wide A, Zakeri S, Carme B, Legrand E, Chevillon C, Ayala FJ, Renaud F, Prugnolle F (2012). Multiple independent introductions of *Plasmodium falciparum* in South America. Proc Natl Acad Sci USA.

