# Supplementary material for: Controlled Human Malaria Infection of Tanzanians by Intradermal Injection of Aseptic, Purified, Cryopreserved Plasmodium falciparum Sporozoites
Source: Am J Trop Med Hyg. 2014 Sep 3;91(3):471–80. doi: 10.4269/ajtmh.14-0119 (PMC4155546; doi:10.4269/ajtmh.14-0119)
Supplement: Supplementary file 1 [file SD3.pdf]

Supplemental Table 1

Results of potency and sporozoite membrane integrity assays (SMIA) on the lot of PfSPZ Challenge used in the clinical trial in Bagamoyo, Tanzania*

Time point	Potency (no. of parasites expressing PfMSP-1/well)	% Viability (sporozoite membrane integrity assay)
Fresh	32.7 ± 1.5	98.2%
Release	29.3 ± 3.1	87.4% ± 5.9%
3 Month	27.3 ± 0.6	84.6% ± 1.9%
6 Month	26.7 ± 1.5	83.6% ± 5.5%
Post-last clinical dose–Oxford[13] 9 Month	26.3 ± 2.5	86.3% ± 6.5%
12 Month	27.3 ± 0.6	86.2% ± 1.3%
Post-last clinical dose–Tanzania (18 month)	24.0 ± 1.7	81.7% ± 2.6%

*Fresh PfSPZ used for the lot of PfSPZ Challenge used in this clinical trial produced 10% more PfMSP-1-expressing parasites in this assay than did PfSPZ that had been cryopreserved for several days (Release). At 18 months, several weeks after inoculation of the last volunteers in Bagamoyo, the PfSPZ had a 27% reduction in potency by this assay as compared with fresh PfSPZ. There was an 11% reduction in the results of the sporozoite membrane integrity of cryopreserved PfSPZ at the time of Release, as compared with fresh PfSPZ. At 18 months, several weeks after inoculation of the last volunteers in Bagamoyo, the PfSPZ had a 17% reduction in the SMIA as compared with fresh PfSPZ.

Supplemental Table 2

Normal ranges at BRTC Laboratory and corresponding modified FDA Guidelines for toxicity grading*

Test	Normal Range	Grade 1	Grade 2	Grade 3	Grade 4
Sodium (meq/l)–hypernatremia	136–146	N/A	146–147	148–150	> 150
Sodium (meq/l)– hyponatremia		132–135	130–131	125–129	< 125
Potassium (meq/l)– hyperkalemia	3.5–5	5.1–5.2	5.3–5.4	5.5–5.6	> 5.6
Potassium (meq/l)– hypokalemia		N/A	3.3–3.4	3.1–3.2	< 3.1
Creatinine (μmol/L)	53–106	107–150	151–177	178–221	> 221 or requires dialysis
Glucose (mmol/L)–hyperglycemia	3.89–6.0	6.01–6.94	6.95–11.1	> 11.1	
Glucose (mmol/L)– hypoglycemia		3.61–3.88	3.05–3.60	2.50–3.04	< 2.50
AST (U/L)	5–34	1.1–2.5 × ULN	2.6–5 × ULN	5.1–10 × ULN	>10 × ULN
ALT (U/L)	0–55	1.1–2.5 × ULN	2.6–5 × ULN	5.1–10 × ULN	>10 × ULN
Bilirubin (μmol/L)	0–6.1	1.1–1.5 × ULN	1.6–2.0 × ULN	2.0–3.0 × ULN	> 3.0 × ULN
Total white blood count × 1,000/μL	3.2–11.7	2.5–3.1	1.5–2.5	1.0–1.5	< 1.0
Neutrophil count × 1,000/μL	2.0–6.9	1.5–1.999	1.0–1.499	0.500–0.999	< 0.500
Lymphocyte count × 1,000/μL	0.87–3.19	0.750–0.869	0.500–0.749	0.250–0.499	< 0.250
Eosinophil count × 1,000/ μL	0–0.7	0.701–1.5	1.501–5.0	> 5.0	
Hemoglobin (g/L)	12.9–18.3	12.5–13.5	10.5–12.4	8.5–10.4	< 8.5
Platelets × 1,000/μL	146–345	125–145	100–124	25–99	< 25

*Guidance for Industry - Toxicity Grading Scale for Healthy Adult and Adolescent Volunteers Enrolled in Preventive Vaccine Clinical Trials – 2007 (http://www.fda.gov/downloads/BiologicsBloodVaccines/GuidanceComplianceRegulatoryInformation/Guidances/Vaccines/ucm091977.pdf). AST = aspartate aminotransferase; ALT = alanine aminotransferase; ULN = upper limit of normal.

Supplemental Table 3

Laboratory abnormalities that developed after administration of normal saline or PfSPZ Challenge from Day 5 through Day 28 post inoculation

	Normal saline controls (*N* = 6) 1st day AE noted* (maximum Grade)†	10,000 PfSPZ (*N* = 12) 1st day AE noted (maximum Grade)*	25,000 PfSPZ (*N* = 12) 1st day AE noted (maximum Grade)*
Increased AST	6 (2), 14 (2)	9 (1), 9 (2), 12 (1), 12 (2)	5 (3), 14 (3), 15 (1), 18 (2)
Increased ALT	14 (3), 15 (1)	9 (2), 12 (1)	5 (2), 14 (2), 15 (1)
Increased Bilirubin		9 (2)	
Increased Creatinine	18 (1)		
Hypoglycemia	5 (2), 28 (1)	15 (1) <u>and</u> 19 (1), 15 (1), 15 (1)	5 (2) <u>and</u> 12 (3)
Hyperglycemia	5 (2)	5 (2), 15 (1)	5 (1), 5 (2), 11 (1), 13 (2), 13 (2), 19 (2)
Leukopenia		18 (1)	
Leukocytosis	14 (1)		
Neutropenia	5 (1) <u>and</u> 18 (1), 6 (1) <u>and</u> 28 (2), 9 (2), 27 (1)	5 (2), 5 (2), 9 (1), 12 (1) <u>and</u> 18 (1), 12 (1), 27 (1), 28 (1)	5 (2), 12 (1), 27 (2)
Lymphopenia	21 (1)	15 (1), 18 (2)	14 (1), 14 (3), 15 (1)
Eosinophilia		5 (1)	
Anemia		28 (1), 28 (1)	11 (1), 15 (2)
Thrombocytopenia	6 (1)	5 (2) <u>and</u> 12 (2), 9 (2), 13 (3), 18 (1)	15 (1)
Proteinuria		5 (3), 5 (2), 12 (2), 12 (2), 28 (3)	5 (2), 9 (2)
Hematuria		28 (2)	

*Each entry represents a specific, different laboratory abnormality.
†For those designated "<u>and</u>", the abnormality occurred, resolved, and recurred.
AE = adverse event; AST = aspartate aminotransferase; ALT = alanine aminotransferase.
